# Modeling and Optimization of Herb-Fortified Fresh Kombucha Cheese: An Artificial Neural Network Approach for Enhancing Quality Characteristics

**DOI:** 10.3390/foods13040548

**Published:** 2024-02-10

**Authors:** Biljana Lončar, Lato Pezo, Mirela Iličić, Katarina Kanurić, Dajana Vukić, Jovana Degenek, Vladimir Vukić

**Affiliations:** 1 Faculty of Technology Novi Sad, University of Novi Sad, Bulevar Cara Lazara 1, 21000 Novi Sad, Serbia; panim@uns.ac.rs (M.I.); stay@uns.ac.rs (K.K.); dajanavukic@uns.ac.rs (D.V.); degenek.9.19.d@uns.ac.rs (J.D.); vukicv@uns.ac.rs (V.V.); 2 Institute of General and Physical Chemistry, Studentski trg 12/V, 11000 Belgrade, Serbia; latopezo@yahoo.co.uk

**Keywords:** antimicrobial potential, antioxidant activity, *Salvia officinalis*, *Thymus serpyllum* L., ANN modeling, optimal formulation, kombucha, fresh cheese, extracts

## Abstract

In this study, an Artificial Neural Network (ANN) model is used to solve the complex task of producing fresh cheese with the desired quality parameters. The study focuses on kombucha fresh cheese samples fortified with ground wild thyme, supercritical fluid extract of wild thyme, ground sage and supercritical fluid extract of sage and optimizes the parameters of chemical composition, antioxidant potential and microbiological profile. The ANN models demonstrate robust generalization capabilities and accurately predict the observed results based on the input parameters. The optimal neural network model (MLP 6-10-16) with 10 neurons provides high r2 values (0.993 for training, 0.992 for testing, and 0.992 for validation cycles). The ANN model identified the optimal sample, a supercritical fluid extract of sage, on the 20th day of storage, showcasing specific favorable process parameters. These parameters encompass dry matter, fat, ash, proteins, water activity, pH, antioxidant potential (TP, DPPH, ABTS, FRAP), and microbiological profile. These findings offer valuable insights into producing fresh cheese efficiently with the desired quality attributes. Moreover, they highlight the effectiveness of the ANN model in optimizing diverse parameters for enhanced product development in the dairy industry.

## 1. Introduction

Artificial Neural Networks (ANNs) are among the most remarkable predictive methods with the ability to learn from examples, with imperfection tolerance, to operate under real-time conditions and to predict non-linear data, making them a regularly used statistical tool in various scientific fields, including cheese production [1,2,3].

According to the literature, ANN has been effectively used for predicting the shelf life of processed cheese [4], vacuum-packed soft cheese [5], French cheeses [6], white brined cheese [7], and Gouda cheese [1].

Furthermore, Horiuchi et al. [8] used an ANN equipped with a culture database to forecast the behavior of the cheese production process. The research revealed that the precise determination of the final process time during the acidification step, which precedes the addition of rennet, is crucial for the successful completion of cheese processing.

On the other hand, Cevoli et al. [9] employed an electronic nose and an ANN approach to categorize Pecorino cheeses based on their ripening time and manufacturing methods. The diverse ANN models, each using different pre-treatment methods, demonstrated varying capabilities in predicting the categories of Pecorino cheeses.

Research conducted by Soto-Barajas et al. [10] predicted the ripening time and milk mixture types in cheeses with varying compositions using ANN. The most effective neural network model for identifying milk mixture types utilized information on fatty acid concentration, achieving 80% accuracy in the training phase and 75% in the validation phase. Another neural network, incorporating near-infrared spectroscopy spectra information, accurately predicted cheese ripening with 100% accuracy in both the training and validation phases.

Ebrahimpour et al. [11] investigated various models for predicting the pH value in fresh cheese production, utilizing laboratory and industrial-scale data in the presence of disturbances. The ANN model, configured with optimal feedback and time intervals using experimental pH data, successfully predicted the pH dynamics of industrial fermentation and provided reliable predictions at both laboratory and industrial scales.

In Santos et al.’s study [12], artificial neural networks and linear discriminant functions were generated using literature data. These models demonstrated the ability to classify 100% of cheeses from various regions based on their physicochemical composition.

All these findings indicate that ANN is a valuable tool for efficient and effective characterization of various cheeses from different regions using readily available physicochemical data.

While the remarkable capabilities of ANN in predicting various parameters of cheese production have been extensively explored, the synergy between predictive modeling and the incorporation of natural plant additives introduces a new dimension in improving the quality and characteristics of cheese products.

Natural plant additives (herbs or their extracts, condiments, vegetables, and other seasonings) are typically flavoring agents included in cheese production to modify its taste and increase its storage shelf life [13,14]. These supplements also change the color of the cheese and enhance its appearance and attractiveness. Furthermore, numerous common herbs traditionally used have both antioxidant and antimicrobial activities [13,15,16]. Herb cheese commonly may include green chili pepper [17], hot pepper [18], Jalapeno red pepper [19], pepper, parsley, dill [20], black peppercorns [21], horseradish [22], ginger, clove, and thyme essential oils [23], black cumin [24], caraway [25] parsley and ginger essential oil [26], tarragon essential oil [27], nutmeg, basil, majoran and oregano essential oil [28], garlic [29], wild onion [30], and tomato powder [31].

**Figure 1:**
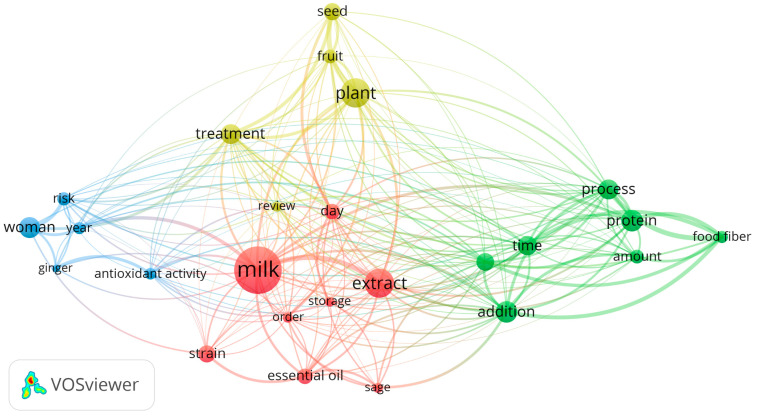
Co-occurrence analysis of herbal cheese meta data abstracts from Scopus.

To identify trends in scientific papers dealing with herbal cheese production, the VOSviewer program was used to represent the author and index keywords. To perform a general analysis, a search was conducted 60 times in the abstracts. As can be seen in Figure 1, co-occurrence analysis of metadata on herbal cheese was distributed in four different groups.

Material and storage-related words were collected in the red cluster (relating to herbal cheese), with the words “milk”, “extract”, “storage” and “essential oil’ being the most frequently mentioned in the summaries analyzed. The green cluster included process parameters applied in herbal cheese production. The most frequent terms in the green cluster were “process”, “addition”, “protein”, “amount”, and “food fiber”. The yellow cluster summarized herbal additives for cheese, and most frequently used terms were “plant”, “seed”, “fruit” and “treatment”. The blue group covered antioxidant activity, ginger addition, year, risk and women as the most frequently used terms.

The size of the circle represents the frequency of occurrence beneath each word. Various colors were employed to illustrate different clusters of highly interconnected keywords, enabling their categorization. The VOSviewer software ver. 1.6.20 was utilized to describe the phrase structure, with data gathered from the Scopus database. Current research usually lacks a comprehensive investigation into the complex relations within herbal cheese production. There is a requirement for in-depth research into the effects of herbal cheese production parameters on the quality and safety of the final products. Moreover, the optimization of herbal cheese production processes using mathematical models for diverse herbal materials and cheese types remain areas that warrant further attention. Overcoming these knowledge gaps is the key to progress in this field and to unlocking the full potential of mathematical modeling in optimizing the performance of herbal cheese production.

Recent research successfully demonstrated the creation of a novel product—kombucha fresh cheese—by employing kombucha inoculum as a non-conventional starter culture. This approach led to a notable reduction in fermentation time, elevated antimicrobial activity and increased total phenols content [32]. Furthermore, kombucha fresh cheese was fortified with sage herbal dust [33] and wild thyme [34], which resulted in changes in the physicochemical properties, antioxidant activity, sensory characteristics, and shelf life of cheese samples.

The quality of cheese during production is influenced by a range of critical parameters [35]. These include factors related to milk composition and quality, the use of cultures and starter bacteria [36], coagulation conditions [37], cutting and stirring of curds [38], draining and pressing methods [39], salting techniques [40], maturation and aging conditions [41], microbial activity [42], pH levels [43], temperature control [44], moisture content, storage conditions, sanitation practices, and careful cheese handling [45]. Managing these parameters is essential for ensuring the consistent production of high-quality cheese.

Obtaining fresh cheese with desirable quality parameters is a challenging modelling task; therefore, in this study, the ANN model was employed to provide reliable predictions and optimize the selected parameters of chemical composition (dry matter, fat, ash, proteins in dry matter, and proteins content), a_w_, pH, antioxidant potential parameters (total phenols (TP), DPPH, ABTS and FRAP) and the selected parameters for microbiological profile (the total number of aerobic mesophilic bacteria, *Escherichia coli*, *Listeria monocytogens*, *Staphylococcus aureus* and lactic acid bacteria) of the tested kombucha fresh cheese samples fortified with ground and supercritical fluid extract of wild thyme, as well as ground and supercritical fluid extract of sage. For developing an artificial neural network model, all data were taken from our previously published research articles, in which we analyzed the produced samples in detail [32,33,34]. In the stated research, kombucha fresh cheeses were produced using preparations from two plants: thyme (*Thymus serpillum*) and Salvia (*Salvia officinalis*). The produced cheeses were intentionally contaminated with the selected pathogenic bacteria in order to examine the influence of enriched cheeses with herbs under such conditions. The ANN modeling and optimization is a logical step in continued research in order to define optimal quality parameters in herb-fortified fresh kombucha cheese production.

## 2. Statistical Analysis

The experimental data underwent chemometrical analysis, including color correlation analysis, principal component analysis (PCA), cluster analysis, and artificial neural network. These analyses were conducted using StatSoft Statistica 10.0^®^ software. Additionally, a color plot diagram was created using R software version 4.0.3 (64-bit), employing the “circle” method with an upper-type configuration.

### 2.1. ANN Modeling

A multi-layer perceptron (MLP) structural model, consisting of three layers (input, hidden, and output) was implemented for modelling the artificial neural network model (ANN) for prognostication the chemical composition (dry matter, fat, ash, proteins in dry matter, and proteins content), a_w_, pH, antioxidant potential (TP, DPPH, ABTS and FRAP) and the microbiological profile (the total number of aerobic mesophilic bacteria, *E. coli*, *L. monocytogens, S. aureus* and lactic acid bacteria) of kombucha fresh cheese samples according to the day of storage, type of herb used (sage and wild thyme), and the type of the cheese sample (KC—kombucha fresh cheese control sample; KG—kombucha fresh cheese with the addition of ground herb, and KSFE—kombucha fresh cheese with the addition of herbal supercritical fluid extracts).

Considering the literature references, the ANN models were widely accepted as comprehensively suitable for the solution of nonlinear problems [3,45,46]. Prior to the ANN model building, input and output variables were standardized to augment the exactness of ANN model’s results. Throughout the iterative process, input data were consistently submitted to the ANN network [47,48]. The Broyden–Fletcher–Goldfarb–Shanno (BFGS) algorithm was employed as an iterative tool for solving unconstrained nonlinear optimization in the course of ANN model building.

**Figure 2:**
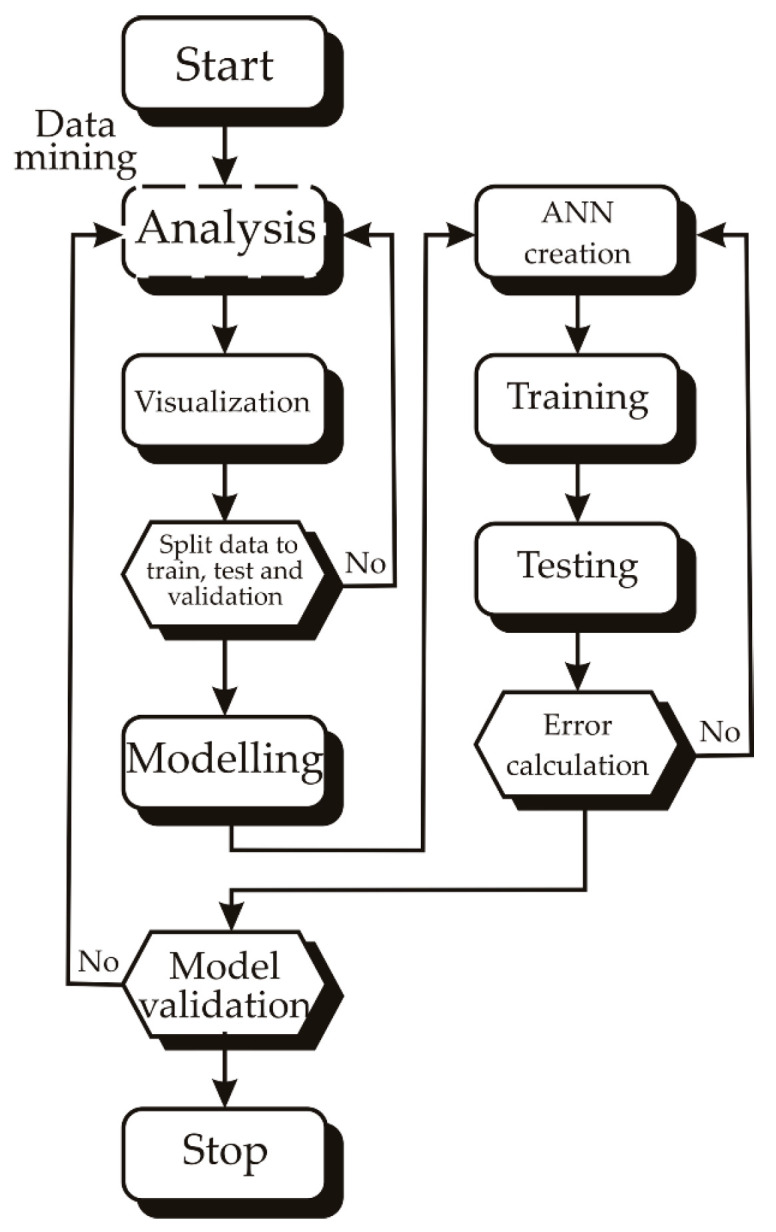
Flowchart of the conducted research.

Figure 2 shows the flowchart of the research conducted with the aim of determining the most appropriate ANN model in terms of predictive ability, but also in terms of the error rate of each model.

The collected data for ANN modelling were randomly partitioned into training, cross-validation, and testing data (with shares of 70%, 15%, and 15% of collected data, respectively). A series of 100,000 different MLP configurations were studied, through the training cycle, by changing the number of neurons in hidden layer (between 5 and 10) applying random preliminary values of weights and biases for the ANN model, and testing different activation functions for the hidden and the output layer (such as hyperbolic tangent, logistic sigmoidal, exponential or identity). Using the identity function, the activation level of the input is passed on directly as the output of the neurons. Logistic uses the logistic sigmoid S-shaped function, with an output in the range from 0 to +1. The hyperbolic tangent function (tanh) is a symmetric S-shaped (sigmoid) function, whose output lies in the range from −1 to +1. It often performs better than the logistic sigmoid function due to its symmetry. Exponential uses the negative exponential activation function.

The optimization setup included the minimization of the square error. It is assumed that the successful training was reached when learning and cross-validation curves approached zero.

The coefficients involved with the hidden layer (weights and biases) were split up in matrices *W*_1_ and *B*_1_. Moreover, coefficients connected to the output layer were combined with matrices *W*_2_ and *B*_2_. It is feasible to describe the neural network models by utilizing matrix record (*Y* is the matrix of the output variables (the dry matter, fat, ash, proteins in dry matter, and proteins content, a_w_, pH, TP, DPPH, ABTS, FRAP, the total number of aerobic mesophilic bacteria, *E. coli*, *L. monocytogens*, *S. aureus* and lactic acid bacteria), f_1_ and f_2_ are transfer functions in the hidden and output layers, accordingly, and *X* is the matrix of input variables (the day of storage, type of herb used (salvia and wild thyme), and the type of the cheese sample (KC—kombucha fresh cheese control sample; KG—kombucha fresh cheese with the addition of ground herb, and KSFE—kombucha fresh cheese with the addition of herbal supercritical fluid extracts [49,50]:

(1) $$Y=f_{1}\left(W_{2}\cdot f_{2}\left(W_{1}\cdot X+B_{1}\right)+B_{2}\right)$$

Weight coefficients in the Artificial Neural Network (ANN) models, represented by elements in matrices *W*_1_ and *W*_2_, as well as vectors *B*_1_ and *B*_2_, were established by determining the ANN model [47]. The widely used BFGS algorithm was employed to ensure convergence and resolve the solution of the nonlinear problem [3].

### 2.2. Global Sensitivity Analysis

Yoon’s interpretation method was utilized to determine the relative influence of the day of storage, type of herb used (sage and wild thyme), and the type of the fresh cheese sample (KC—kombucha fresh cheese control sample; KG—kombucha fresh cheese with the addition of ground herb, and KSFE—kombucha fresh cheese with the addition of herbal supercritical fluid extracts) on the selected parameters of chemical composition, antioxidant potential, and microbiological profile of kombucha fresh cheese. This calculation was performed according to the weight coefficients of the erected ANN model [40].

The provided equation was employed to assess the direct impact of the input parameters on the output variables, considering the weighting coefficients embedded within the Artificial Neural Network (ANN) model [50]:

(2) $${RI}_{ij}\left(\%\right)=\sum_{k=0}^{n}\left(w_{ik}\cdot w_{kj}\right)/\left(\sum_{i=0}^{m}\left|\sum_{k=0}^{n}\left(w_{ik}\cdot w_{kj}\right)\right|\right)\cdot 100\%$$

where *w*—presents the weights of the ANN model, *i*—input variable, *j*—output variable, *k*—hidden neuron, *n*—number of hidden neurons, *m*—number of inputs.

### 2.3. The Accuracy of the Model

The statistical validation of the formulated non-linear models was investigated employing standard computational tests, which encompassed the coefficient of determination (r^2^), reduced chi-square (χ^2^), mean bias error (MBE), root-mean-square error (RMSE), and mean percentage error (MPE). These metrics were evaluated using the following equations [48]:

(3) $$\chi^{2}=\sum_{i=1}^{N}{\left(x_{\exp,i}-x_{pre,i}\right)}^{2}/\left(N-n\right)$$

(4) $$RMSE={\left[\frac{1}{N}\cdot \sum_{i=1}^{N}{\left(x_{\exp,i}-x_{pre,i}\right)}^{2}\right]}^{\frac{1}{2}}$$

(5) $$MBE=\frac{1}{N}\cdot \sum_{i=1}^{N}\left(x_{pre,i}-x_{\exp,i}\right)$$

(6) $$MPE=\frac{100}{N}\cdot \sum_{i=1}^{N}\left(\frac{\left|x_{pre,i}-x_{\exp,i}\right|}{x_{\exp,i}}\right)$$

where *x*_exp,*i*_ were collected values and *x_pre,i_* were the model anticipated values; *N* and *n* are the number of observations and constants, accordingly.

## 3. Results and Discussion

### 3.1. Correlation Analysis

**Figure 3:**
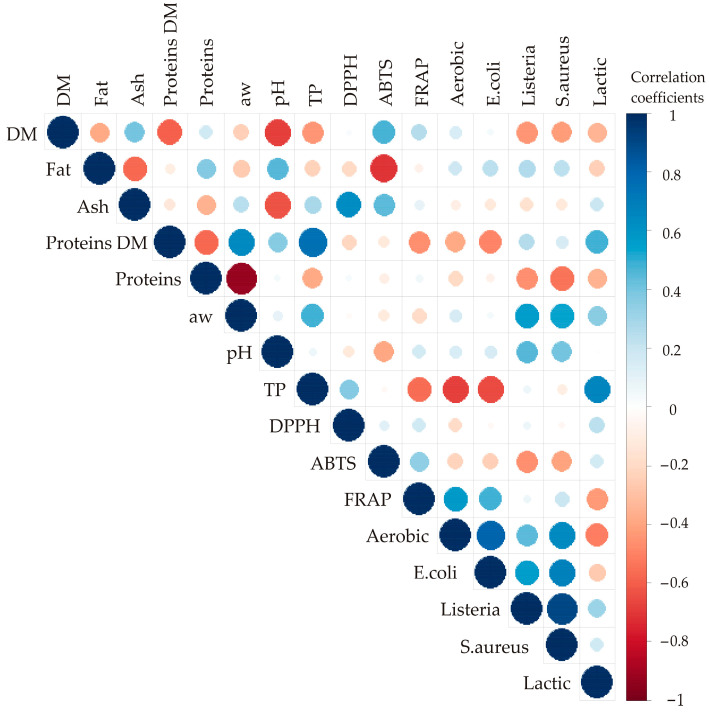
Color correlation graph between observed responses for chemical composition, antioxidant potential, and microbiological profile of kombucha fresh cheese samples.

Correlation analysis revealed statistically significant associations (*p* ≤ 0.05) among various responses in the examined kombucha fresh cheese samples (Figure 3). The correlation coefficients specify the size and color of the circles presented in Figure 3. A blue circle implies a positive correlation, while a red circle indicates a negative correlation between observed responses. Additionally, the size of the circle increases with the absolute value of the correlation coefficient [51]. The highest positive correlations were found between *Listeria monocytogenes* and *Staphylococcus aureus* (r = 0.909; *p* ≤ 0.001), and also between *Escherichia coli* and Aerobic mesophilic bacteria (r = 0.808, *p* ≤ 0.001) and *Escherichia coli and Staphylococcus aureus* (r = 0.689; *p* ≤ 0.001). These results indicate that antimicrobial activity of kombucha fresh cheese is not selective and has a similar impact on all investigated microorganisms. On the other hand, the highest negative correlations were observed between the content of total proteins (%) and a_w_ (r = −0.922, *p* ≤ 0.001). This is the consequence of amphipathic structure of the casein micelle, that are able to bind water on its surface and lower the a_w_ value of the cheese [52].

### 3.2. PCA and Cluster Analysis

**Figure 4:**
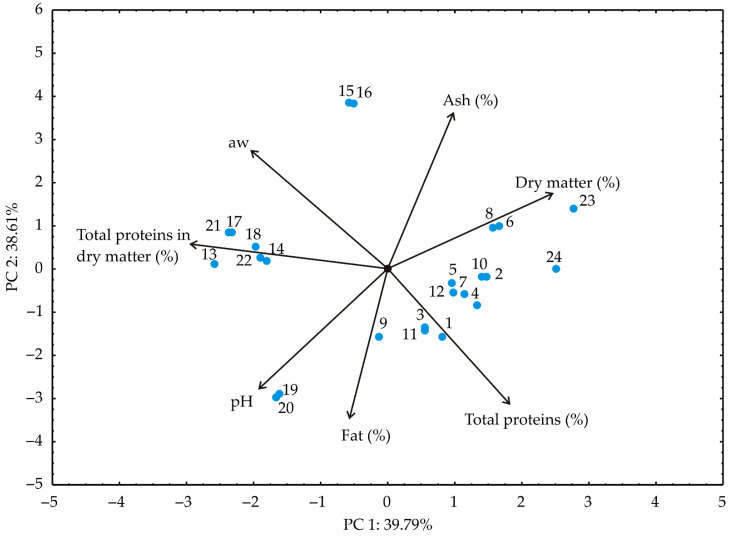
The PCA biplot diagram of the relationships among observed responses for chemical composition of fresh kombucha cheese.

**Figure 5:**
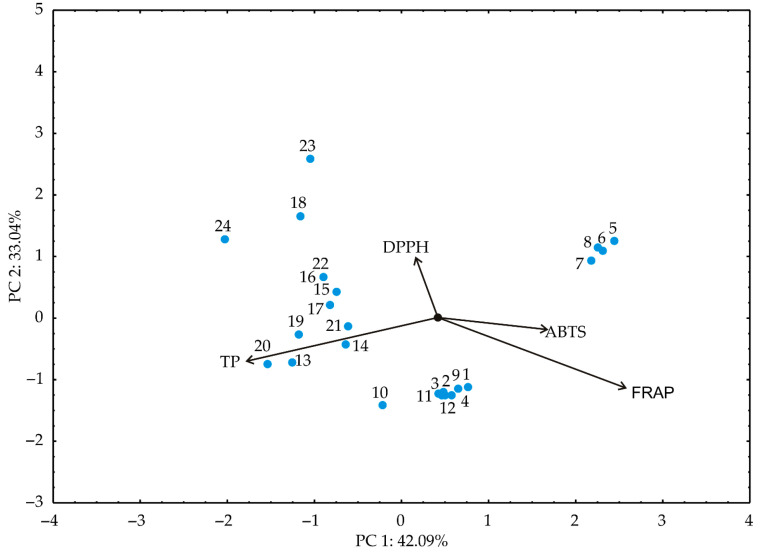
The PCA biplot diagram of the relationships among observed responses for antioxidant potential of fresh kombucha cheese samples.

**Figure 6:**
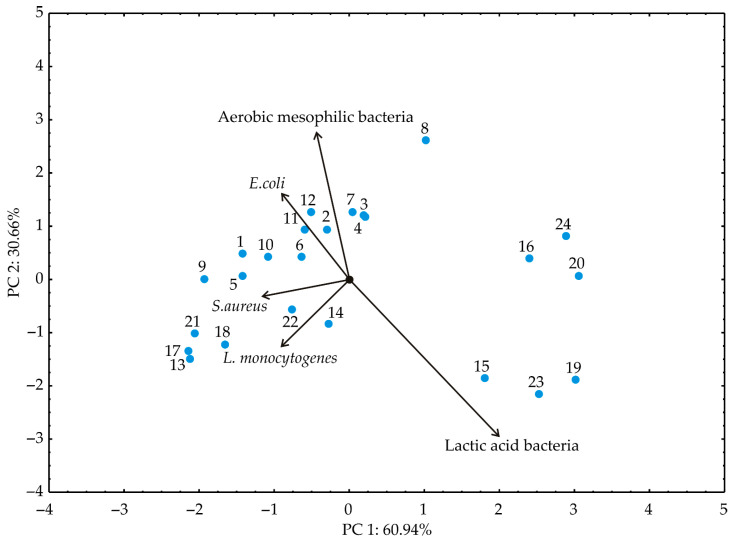
The PCA biplot diagram of the relationships among observed responses for microbiological profile of fresh kombucha cheese samples.

Principal component analysis (PCA) aided in discerning patterns within the analyzed data by providing insights into identifying variables that exhibit similar behavior [53]. In the PCA plot, closely positioned dots signify similarities in the patterns representing these samples, according to experimental plan presented in Table 1. The orientation of vectors in factor space indicates an increasing trend of these factors. Meanwhile, the size of the vectors is proportional to the squared correlation among the variables. The angles between corresponding variables reflect the magnitude of their correlations, with sharper angles indicating higher correlations [54]. Utilizing the experimental findings, samples are marked as shown in Table 1; principal component analysis (PCA) was conducted as shown in Figure 4, Figure 5 and Figure 6.

The PCA biplot of the relationships among the dry matter, fat, ash, proteins in dry matter, proteins content, a_w_, and pH of the tested kombucha fresh cheese samples revealed that the first two principal components explained 78.40% of the total variance in the observed parameters, as shown in Figure 4. Based the results of the PCA, the dry matter, total proteins in dry matter (%), a_w_, and pH, (22.46%, 26.93%, 18.30%, and 14.81% of the total variance, based on correlations, respectively) showed a positive influence on the first principal coordinate, while fat (−22.88%), ash (−23.92%), and total proteins (−18.79%), negatively contributed to the calculation of the PC2. It is notable grouping of the samples fortified with salvia after 0 and 10 days of storage at the negative values of the PC1. These samples are characterized by their total protein content in dry matter.

The PCA biplot of the relationships among the antioxidant potential parameters (TP, DPPH, ABTS and FRAP) of the tested kombucha fresh cheese samples revealed that the first two principal components explained 75.04% of the total variance in the observed parameters, as shown in Figure 5.

Produced chesses were grouped by the type and form of the supplement, rather than the day of storage. Based the results of the PCA, the TP (−40.82% of the total variance, based on correlations) showed a negative influence on the first principal coordinate, while FRAP (45.62%) positively contributed to the calculation of the PC1. On the other hand, DPPH and ABTS positively influenced PC 2 (56.07% and 22.99% of the total variance, based on correlations, respectively).

The PCA biplot of the relationships among the selected responses for microbiological profile (aerobic mesophilic bacteria, *E. coli*, *S. aureus*, *L. monocytogenes*, and Lactic acid bacteria) of the tested kombucha fresh cheese samples revealed that the first two principal components explained 91.60% of the total variance in the observed parameters, as shown in Figure 5.

In contrast to the antioxidant potential parameters, the samples were grouped according to antimicrobial activity by day of storage and not by supplement type and form. According to the results of the PCA, Aerobic mesophilic bacteria, namely *E. coli*, *S. aureus*, and *L. monocytogenes* (−23.79%, −25.88%, −22.05 and −27.74% of the total variance, based on correlations, respectively), showed a negative influence on the first principal coordinate. On the other hand, lactic acid bacteria negatively influenced PC 2 (−59.87% of the total variance, based on correlations).

**Figure 7:**
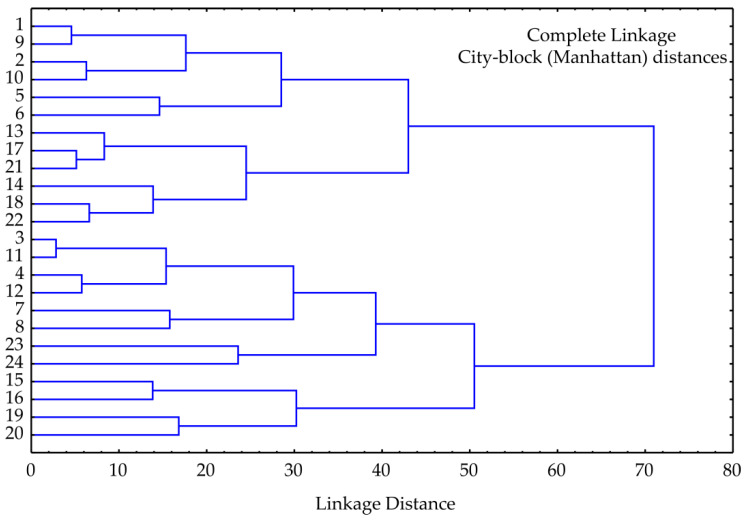
The cluster analysis of the observed samples representing the linkage distances among the samples.

The results of cluster analysis performed for the selected parameters of chemical composition (dry matter, fat, ash, proteins in dry matter, and protein content), aw, pH, antioxidant potential parameters (TP, DPPH, ABTS and FRAP), and the selected parameters for microbiological profile (the total number of aerobic mesophilic bacteria, *E. coli*, *L. monocytogens*, *S. aureus* and lactic bacteria) of the tested kombucha fresh cheese samples are given in Figure 7. The cluster analysis dendrogram revealed two main separate clusters, and four sub clusters. The first cluster contained samples 1, 9, 2,10, 5, 6, 13, 17, 21, 14, 18, and 22. On other hand, the second one involved samples 3, 11, 4, 12, 7, 8, 23, 24, 15, 16, 19, and 20. Therefore, the samples are divided into two groups according to the day of storage. The first cluster contains samples after 0 and 10 days of storage, while the second cluster contains samples after 20 and 30 days of storage. The linkage distance (illustrated on the abscissa axis) between the main clusters was nearly 70.

### 3.3. Artificial Neural Network Modeling

**Figure 8:**
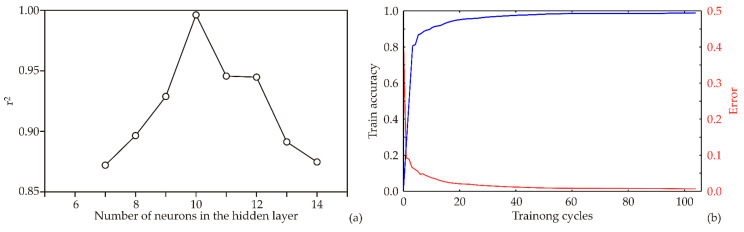
ANN calculation: (**a**) The dependence of the r^2^ value of the number of neurons in the hidden layer in the ANN model, (**b**) training results per epoch.

In this study an ANN model was developed, with its structure and performance heavily reliant on initial assumptions regarding matrix parameters (biases and weight coefficients). These parameters play a pivotal role in molding the ANN to fit experimental data accurately. Moreover, the number of neurons in the hidden layer can influence the performance of the model. To counteract potential issues, each topology underwent 100,000 runs to eliminate any random correlation from initial assumptions and random weight initialization. This meticulous approach resulted in the ANN model achieving its highest r^2^ value during training with nine hidden neurons (Figure 8a).

The ANN model underwent training for 100 epochs, highlighting its training results in Figure 8b, specifically the train accuracy and error (loss). The accuracy increased steadily with the number of training cycles until it plateaued around the 30–50th epoch. Going beyond 50 epochs might lead to significant overfitting, while stopping at 50 epochs was sufficient to attain high model accuracy without risking overfitting (refer to Figure 2).

The acquired optimal neural network models showed good generalization capabilities for the foreseen collected data, and could be used to accurately predict the observed outputs, based on the input parameters. The required number of neurons for the ANN model was 10 (network MLP 6-10-16) in order to obtain the highest values of r^2^ (the r^2^ values for prediction of output variables were 0.993, 0.992 and 0.992, for training, testing and validation cycles, respectively), Table 2.

Table 3 presents the elements of matrix *W*_1_ and vector *B*_1_ (presented in the bias row). Table 4 presents the elements of matrix *W*_2_ and vector *B*_2_ (bias) for the hidden layer used for calculation within the ANN model.

**Figure 9:**
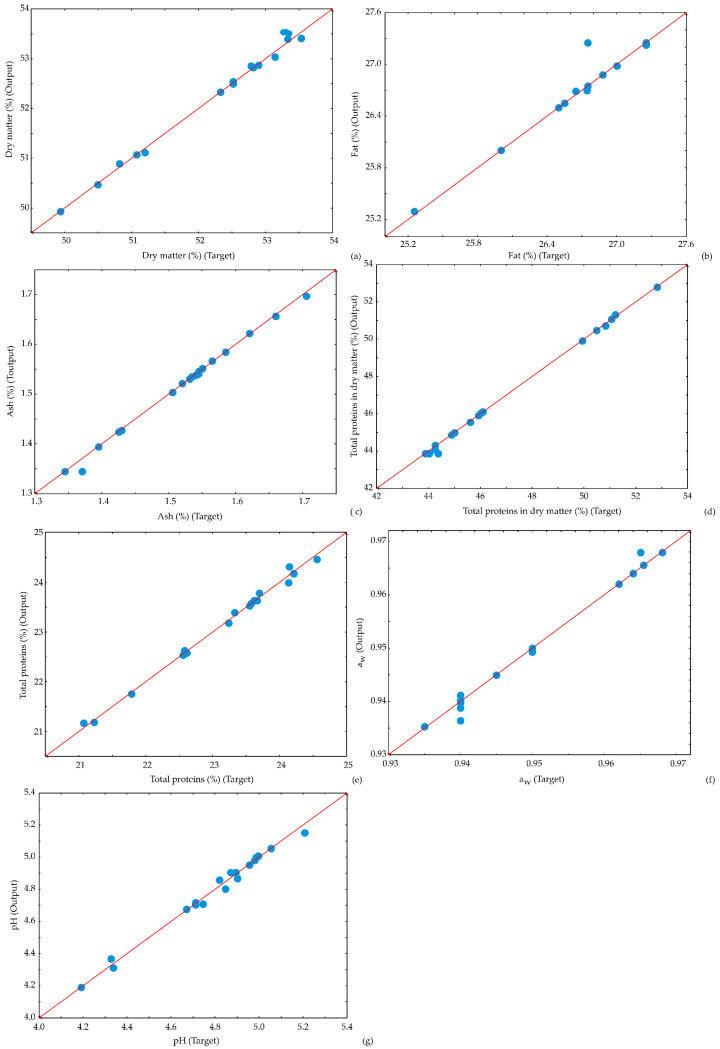
Comparison between experimentally obtained and ANN model predicted values of (**a**) dry matter (**b**) fat content, (**c**) ash content, (**d**) total proteins in dry matter, (**e**) total proteins, (**f**) a_w_, and (**g**) pH.

**Figure 10:**
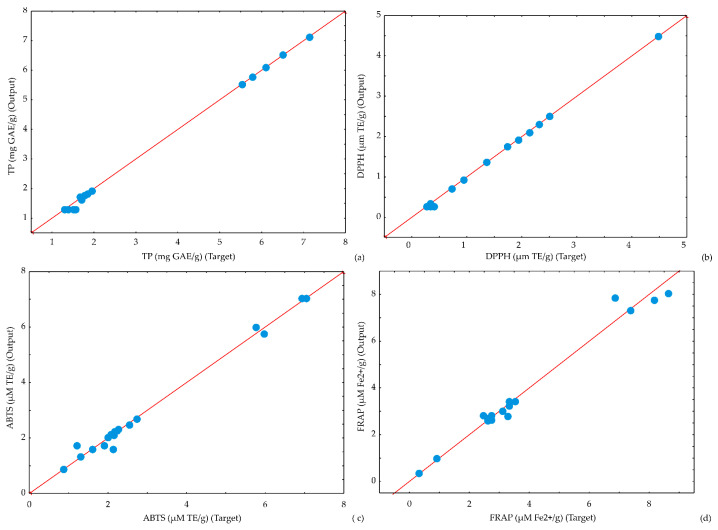
Comparison between experimentally obtained and ANN model predicted values of (**a**) TP (**b**) DPPH, (**c**) ABTS, and (**d**) FRAP.

**Figure 11:**
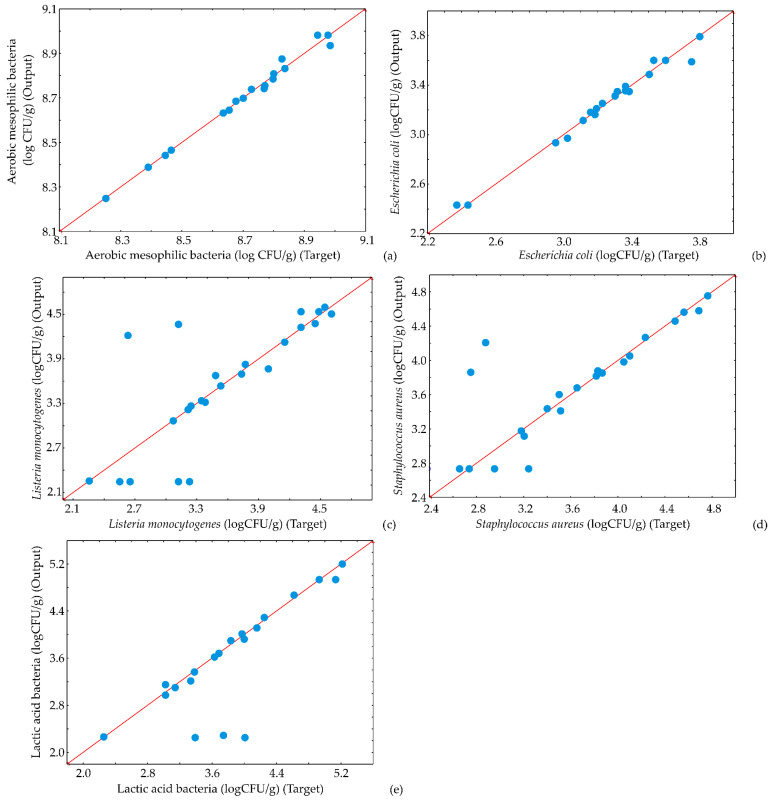
Comparison between experimentally obtained and ANN model predicted values of (**a**) aerobic mesophilic bacteria (**b**) *E. coli*, (**c**) *L. monocytogenes*, (**d**) *Staphilococcus aureus*, and (**e**) lactic acid bacteria.

The potential of the ANN model to predict observed parameters is presented by scatter plots (Figure 9, Figure 10 and Figure 11). Figure 9, Figure 10 and Figure 11 display the experimentally estimated and ANN model-predicted values of the observed responses for kombucha fresh cheese samples, suggesting that the ANN model correctly predicted experimental variables.

The elevated levels of pathogenic bacteria presented in Figure 11 were deliberately induced for experimental purposes, as stated in our previously published papers [32,33,34]. The cheese samples were intentionally contaminated to examine the specific influence of herbs under such conditions. This approach allowed exploration of the potential effects of herbs and herbal extracts on the microbiological profile of kombucha fresh cheese, providing insights into their antimicrobial properties or other relevant interactions.

### 3.4. The Accuracy of the Model

To numerically verify the displayed models accuracy coefficient of determination (r^2^), reduced chi-square (χ^2^), mean bias error (MBE), root-mean-square error (RMSE), and mean percentage error (MPE) were calculated, as shown in Table 5. The results show that the ANN models had a minor lack of fit tests, which implies that the models satisfactorily predicted the values of the analyzed parameters.

However, the importance of conducting external validation using independent datasets or real-world experiments to ascertain the robustness and generalizability of these findings should be highlighted, while also acknowledging the potential limitations in fully addressing long-term implications or changes within the study, thereby underscoring the imperative for further research in this area.

### 3.5. Global Sensitivity Analysis—Yoon’s Interpretation Method

**Figure 12:**
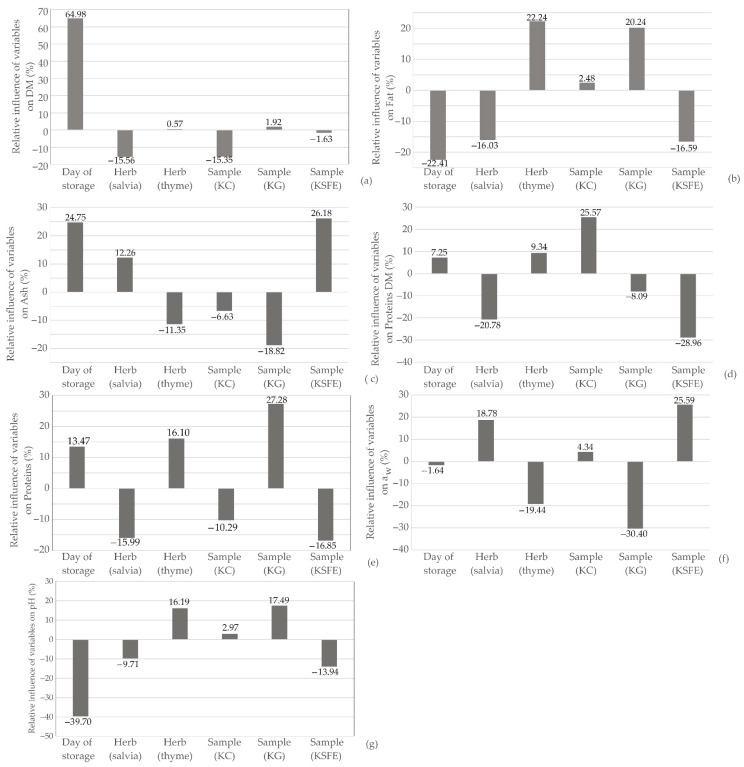
Relative importance of the day of storage, herb selection and kombucha fresh cheese sample type on: (**a**) dry matter content, (**b**) fat content, (**c**) ash content, (**d**) content of proteins in dry matter, (**e**) protein content, (**f**) a_w_ value, and (**g**) pH value.

The influence of input variables on the relative importance of the dry matter content, fat content, ash content, content of proteins in dry matter, protein content, a_w_ value, and pH value for ANN model, is illustrated in Figure 12. In Figure 12, the storage duration emerged as the most influential factor affecting dry matter content, with a significant positive impact of approximately +64.98% (Figure 12a). Additionally, the day of storage played a pivotal role in pH and fat content, but the impact was contradictory, accounting for −39.70% and −22.41% of relative importance, respectively (Figure 12g). The introduction of sage had adverse effects on dry matter (−15.56%), fat (−16.03%), proteins in dry matter (−20.78%), protein content (−15.99%), and pH (−9.71%), while positively impacting ash content (+12.26%) and water activity (+18.78%). Conversely, the inclusion of wild thyme exhibited a positive influence on fat (+22.24%), proteins (+16.10%), and pH (+16.19%), while negatively affecting ash (−11.35%) and water activity (−19.44%). Regarding cheese sample preparation, samples with ground herbs positively influenced proteins, while those with the addition of herbal supercritical fluid extracts positively affected ash content and water activity value.

**Figure 13:**
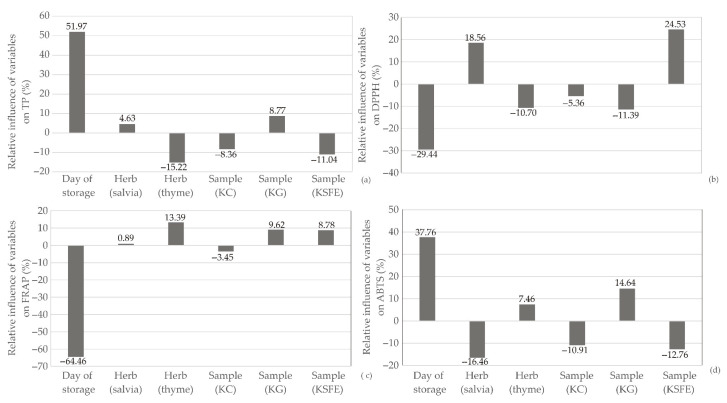
Relative importance of the day of storage, herb selection and kombucha fresh cheese sample type on: (**a**) TP, (**b**) DPPH, (**c**) FRAP, and (**d**) ABTS.

Figure 13 illustrates the impact of input variables on the relative importance of TP, DPPH, FRAP, and ABTS. Notably, the day of storage emerged as the most influential parameter, positively affecting TP (+51.97%) and ABTS (+37.76%), while simultaneously exerting a negative influence on DPPH (−29.44%) and FRAP (−64.46%). Other input parameters demonstrated significantly lower effects on the observed antioxidant potential parameters of kombucha fresh cheese samples. This implies that, within the study’s scope, the day of storage predominantly shapes the antioxidant characteristics of the cheese. The finding that the day of storage significantly influences the antioxidant potential of kombucha fresh cheese aligns with common practices in food science [55]. The distinct effects of the day of storage on different antioxidant parameters (TP, DPPH, FRAP, and ABTS) reflect the complex nature of these compounds [56].

**Figure 14:**
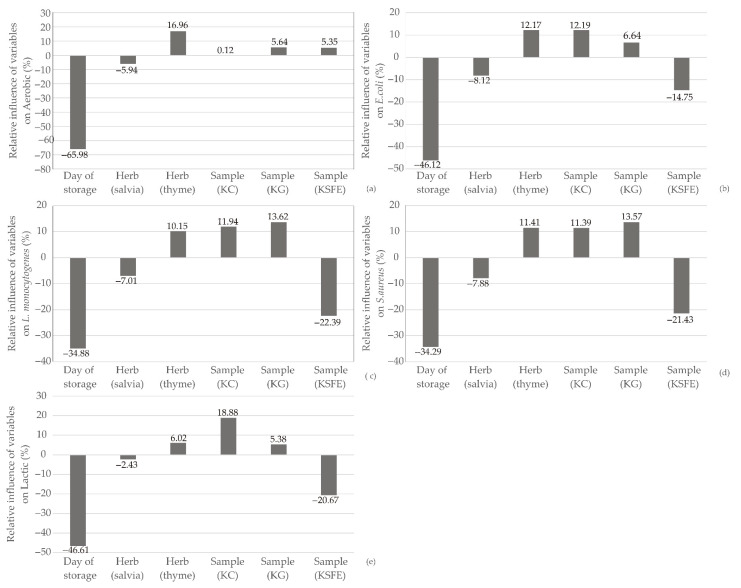
Relative importance of the day of storage, herb selection and kombucha fresh cheese sample type on: (**a**) aerobic mesophilic bacteria, (**b**) *E. coli*, (**c**) *L. monocytogenes*, (**d**) *S. aureus*, and (**e**) lactic acid bacteria.

The influence of input variables on the relative importance of aerobic mesophilic bacteria, *E. coli*, *L. monocytogenes*, *S. aureus*, and lactic acid bacteria for ANN model, was given in Figure 14. Significantly, the day of storage stands out as the most influential parameter, exerting a negative impact on the observed responses of aerobic mesophilic bacteria (−66.98%), *E. coli* (−46.12%), *L. monocytogenes* (−34.88%), *S. aureus* (−34.29%), and lactic acid bacteria (−46.61%). The research by Tiwari et al. [57] underlined that the post-processing storage conditions at the retail level are critical factors affecting *L. monocytogenes* concentration. The negative impact observed in the ANN model underscores the need for careful consideration of storage parameters to mitigate potential risks associated with the proliferation of undesirable microorganisms [57]. The integration of ANN modeling with a focus on the day of storage provides valuable insights into the dynamic nature of microbiological responses in kombucha fresh cheese [58,59].

### 3.6. Multi-Objective Optimization of the Outputs of the ANN

The challenges associated with Artificial Neural Network (ANN) models in real-world applications include dependency on data quality and quantity, susceptibility to over fitting and under fitting, lack of interpretability due to their “black-box” nature, high computational resource and time demands, sensitivity to hyper parameters, potential generalization issues, limited causality inference capabilities, and risks of biased predictions [60,61]. Firstly, their accuracy is heavily contingent upon the quality and quantity of input data, and they are susceptible to overfitting or underfitting, hindering their ability to generalize to new data [62]. The “black-box” nature of ANNs makes it challenging to interpret the relationships between inputs and outputs [63]. Additionally, the training and optimization of complex ANN models demand significant computational resources and time [64]. Sensitivity to hyperparameters necessitates careful tuning, and issues with generalization may arise if the training dataset lacks representativeness [65]. While ANNs can capture correlations, their limited ability to infer causality requires additional analysis. Furthermore, the models are sensitive to initial conditions during training, posing a challenge in achieving consistent outcomes. The risk of biased predictions due to biased training data and the need for specialized expertise in machine learning and neural networks contribute to the overall limitations of ANNs. Keeping in mind all the listed ANN limitations, the optimization of the ANN outputs was performed using results presented in Table 2 and Table 3, applied in Equation (1). One of the main goals in this investigation was to optimize DM, Fat, Ash, Proteins DM, Proteins, a_w_, pH, TP, DPPH, ABTS, FRAP, aerobic mesophilic bacteria, *E. coli, L. momocytogens, S. aureus*, and lactic acid bacteria, simultaneously, using the ANN model by changing the input variables (day of storage, herb selection and kombucha fresh cheese sample type). These numerical tasks were solved using the MOO calculation in Matlab. The MOO procedure was defined to find the best combinations of process parameters by optimizing the output variables in the ANN model. The number of generations reached 446 for ANN model, while the size of the population was set to 100 for each input variable. The number of points on the pareto front was 2.

In the realm of ANN model optimization, the results culminated in the identification of an optimal sample—specifically, the one with supercritical fluid extract of sage, stored on the 20th day. This optimal sample exhibited a finely tuned composition, with dry matter at 53.535%, fat at 25.25%, ash at 1.70%, proteins dm at 43.993%, proteins at 24.550%, with aw 0.936 and pH 4.190. Additionally, it showcased desirable values for total phenols (TP), DPPH, ABTS, FRAP, and microbiological components, including Aerobic bacteria, *E. coli*, *L. monocytogenes*, *S. aureus*, and lactic acid bacteria (TP 7.15 mgGAE/g, DPPH 4.482 μM TE/g, ABTS 7.031 μM TE/g, FRAP 0.409 μM TE/g, Aerobic bacteria 6.975 logCFU/g, *E.coli* 2.370 logCFU/g, *L. monocytogenes* 3.115 logCFU/g, *S. aureus* 2.650 logCFU/g, and lactic acid bacteria 6.770 logCFU/g).

This optimization process pinpointed an optimal configuration for the kombucha fresh cheese and underscored the intricate relationships and trade-offs inherent in the various quality parameters. The meticulous tuning of these variables serves as a blueprint for practitioners and researchers, offering a pathway toward achieving the desired characteristics in kombucha fresh cheese production. Achieving the preferable composition of fresh cheese formulation ensures a desirable sensory acceptance by consumers.

Furthermore, the integration of advanced computational techniques, such as Multi-Objective Optimization (MOO) alongside Artificial Neural Network (ANN) models, showcases the evolving landscape of optimization methodologies in the dairy industry. This systematic approach to parameter optimization provides insights into the complex relationships among various quality parameters and sets a framework for future investigations and enhancements in cheese production processes.

## 4. Conclusions

In conclusion, the comprehensive investigation of kombucha fresh cheese, involving correlation analysis, Principal Component Analysis, Cluster analysis, and Artificial Neural Network models, revealed significant associations and provided a multifaceted assessment of quality parameters. Positive correlations among microbiological components and negative correlations between total proteins and water activity were identified. PCA elucidated the diverse impacts of different parameters on variance, specifically in chemical composition, antioxidant potential, and microbiological profiles. The developed ANN models demonstrated robust predictive capabilities. The highlighted optimal sample, kombucha fresh cheese with addition of supercritical fluid extract of sage, KSFE, on the 20th day of storage showcased specific attributes for quality optimization.

Furthermore, the sensitivity analysis emphasized the pivotal role of the day of storage and the influence of herbal additives like sage and wild thyme. These insights underscore the need to carefully consider storage conditions and ingredient choices to achieve desired product characteristics. The study’s findings contribute valuable knowledge for enhancing kombucha fresh cheese production, offering practical guidance for the dairy industry and researchers seeking to refine processes and improve overall product quality.

Future research could delve into investigating long-term storage effects interactions among ingredients, consumer sensory evaluation, optimization of production processes, microbial dynamics, scale-up studies, market trends analysis, and environmental impact to enhance kombucha fresh cheese production and contribute to the dairy industry’s advancement. The study’s findings offer practical guidance for refining processes and improving overall product quality.

## Figures and Tables

**Table 1:** Experimental design—samples of kombucha fresh cheeses used for the analysis fortified with different herbal preparations: KC—kombucha fresh cheese control sample; KG—kombucha fresh cheese with the addition of ground herb, and KSFE—kombucha fresh cheese with the addition of herbal supercritical fluid extracts [32,33,34].

No	Herb	Sample	Day of Storage
1	thyme	KC	0
2	thyme	KC	10
3	thyme	KC	20
4	thyme	KC	30
5	thyme	KG	0
6	thyme	KG	10
7	thyme	KG	20
8	thyme	KG	30
9	thyme	KSFE	0
10	thyme	KSFE	10
11	thyme	KSFE	20
12	thyme	KSFE	30
13	salvia	KC	0
14	salvia	KC	10
15	salvia	KC	20
16	salvia	KC	30
17	salvia	KG	0
18	salvia	KG	10
19	salvia	KG	20
20	salvia	KG	30
21	salvia	KSFE	0
22	salvia	KSFE	10
23	salvia	KSFE	20
24	salvia	KSFE	30

**Table 2:** Artificial neural network model summary (performance and errors), for training, testing, and validation cycles.

	Performance			Error			Training Algorithm	Error Function	Activation	
Net. Name	Train.	Test	Valid.	Train.	Test	Valid.			Hidden	Output
MLP 6-10-16	0.993	0.992	0.992	0.112	0.110	0.099	BFGS 895	SOS	Tanh	Logistic

Net.—Artificial Neural Network, Train.—training cycle, Test—testing cycle, Valid.—validation cycle.

**Table 3:** The weight coefficients and biases *W*_1_ and *B*_1_ for ANN model.

	1	2	3	4	5	6	7	8	9	10
Day of storage	5.946	−74.377	−5.191	−0.123	35.947	19.789	69.472	18.844	11.077	−9.741
Herb (salvia)	−22.188	19.571	2.858	−0.875	−23.021	−1.460	−20.183	−24.839	24.732	−0.049
Herb (thyme)	23.436	−8.728	−1.138	0.791	23.777	−8.585	5.219	17.235	−22.868	3.855
Sample (KC)	−0.108	−7.028	3.362	22.847	−16.967	4.949	11.619	30.853	−28.909	−0.042
Sample (KG)	1.287	−3.620	−2.503	−21.715	8.757	4.618	−37.262	−22.961	22.947	−0.295
Sample (KSFE)	0.132	21.571	0.872	−1.102	9.015	−19.489	10.619	−15.595	7.802	4.176
Bias	1.441	10.877	1.697	−0.082	0.821	−10.059	−15.175	−7.666	1.981	3.787

**Table 4:** The weight coefficients and biases *W*_2_ and *B*_2_ for ANN model.

	1	2	3	4	5	6	7	8	9	10	Bias
DM	58.183	8.481	32.653	−42.411	−16.508	26.829	35.997	29.942	54.881	−4.307	0.671
Fat	−7.297	−31.416	70.373	−66.801	44.395	−34.262	−97.084	50.442	−64.959	−79.962	−1.562
Ash	−5.044	−7.549	−9.331	14.156	−10.769	−49.339	20.769	−12.630	13.787	−41.212	1.185
Proteins DM	−35.584	−2.777	39.702	−54.536	3.466	47.129	−5.042	33.668	−52.901	10.158	−2.158
Proteins	−20.417	−3.559	7.086	−9.984	25.723	11.569	−13.381	5.889	−10.779	3.842	−0.324
a_w_	35.435	16.330	−28.452	38.750	−51.027	−33.065	45.172	−18.905	36.649	−6.693	4.102
pH	−1.433	14.026	11.830	−17.768	25.875	18.149	−44.432	33.513	−16.784	6.469	−0.361
TP	−73.083	−36.334	−15.210	−21.810	19.576	21.696	8.571	−25.220	−22.743	4.998	0.670
DPPH	−18.678	34.277	−50.497	33.291	3.410	−50.724	5.340	17.083	34.110	−28.422	−1.852
ABTS	17.065	0.087	17.405	−23.008	1.717	18.474	3.723	14.220	13.383	3.014	−2.949
FRAP	3.087	6.057	−1.503	2.053	3.001	−1.930	−4.765	5.701	3.037	−0.763	−0.582
Aerobic	65.168	59.848	7.543	−10.703	2.055	3.993	−31.010	23.972	−10.132	−2.797	0.059
*E. coli*	16.318	2.112	4.819	−6.670	−10.296	3.710	−7.232	3.367	−6.008	−0.189	0.417
*L. monocytogenes*	3.493	−3.286	4.604	−6.459	−3.850	4.743	−11.311	2.547	−10.371	0.625	1.358
*S. aureus*	5.659	−4.874	6.536	−9.038	−5.145	4.751	−15.700	3.467	−14.378	−0.910	0.171
Lactic	7.317	−1.893	1.916	−2.428	−8.363	0.305	−4.583	0.212	−4.536	−1.266	0.608

**Table 5:** The “goodness of fit” tests for the developed ANN model.

	χ^2^	RMSE	MBE	MPE	SSE	AARD	r^2^
DM	0.080	0.094	−0.028	0.128	0.146	1.205	0.994
Fat	0.128	0.119	−0.028	0.146	0.242	0.707	0.936
Ash	0.000	0.006	0.002	0.202	0.001	0.054	0.996
Proteins DM	0.170	0.137	0.035	0.166	0.317	1.365	0.998
Proteins	0.045	0.071	−0.003	0.232	0.089	0.914	0.995
a_w_	0.000	0.001	0.000	0.072	0.000	0.012	0.990
pH	0.006	0.027	0.001	0.418	0.013	0.361	0.990
TP	0.083	0.096	0.042	3.361	0.134	0.987	0.999
DPPH	0.023	0.051	0.025	7.456	0.036	0.552	0.999
ABTS	0.350	0.197	−0.005	5.905	0.699	2.390	0.990
FRAP	0.953	0.325	−0.005	6.407	1.905	3.790	0.980
Aerobic	0.856	0.534	−0.236	3.468	5.508	5.868	0.698
*E. coli*	0.482	0.401	−0.169	12.928	3.172	4.612	0.867
*L. momocytogens*	0.777	0.509	−0.022	8.927	6.208	7.013	0.610
*S. aureus*	0.438	0.382	−0.085	6.110	3.328	4.477	0.703
Lactic	6.838	1.510	0.681	13.734	43.561	17.815	0.11

## Data Availability

The original contributions presented in the study are included in the article, further inquiries can be directed to the corresponding author.
